# Using MediaPipe to track upper-limb reaching movements after stroke: a proof-of-principle study

**DOI:** 10.1186/s12984-025-01808-4

**Published:** 2025-11-25

**Authors:** Vaidehi Wagh, Matthew W. Scott, Justin W. Andrushko, Christina B. Jones, Beverley C. Larssen, Lara A. Boyd, Sarah N. Kraeutner

**Affiliations:** 1 Neuroplasticity, Imagery, and Motor Behaviour Laboratory, Department of Psychology, University of British Columbia, BC V1V 1V7 Kelowna, Canada; 2 Motor Cognition, Neuroscience and Behavior Lab, Department of Kinesiology & Sport Management, Texas A&M University, College Station, TX USA; 3 Department of Sport, Exercise and Rehabilitation, Faculty of Health and Life Sciences, Northumbria University, Newcastle-upon-Tyne, Tyne and Wear UK; 4 Graduate Program in Rehabilitation Sciences, University of British Columbia, Vancouver, BC Canada; 5 Department of Physical Therapy, University of British Columbia, Vancouver, BC Canada; 6 Djavad Mowafaghian Centre for Brain Health, University of British Columbia, Vancouver, BC Canada

**Keywords:** Motion tracking, Markerless pose estimation, Artificial intelligence, Motor impairment, Kinematics

## Abstract

Emerging work supports the use of artificial intelligence-based markerless motion capture systems to complement standardized clinical measures when assessing post-stroke motor recovery. MediaPipe Pose Landmarker is an open-sourced, machine learning tool, requiring only one camera, which can be used to track upper limb movements and quantify kinematics. Here we aimed to test the use-case of MediaPipe Pose Landmarker in tracking upper limb movements after stroke in a 2-dimensional cartesian coordinate space. Participants (*N* = 7, > 2 months after stroke, upper extremity portion of the Fugl-Meyer Assessment (FMA-UE) range of 40–66) engaged in five sessions of a previously established, semi-immersive, gamified reaching task, involving movements of the hand and arm. Movements at four time points (first and last block of session 1 and 5) were captured by a video camera, with videos processed through the MediaPipe Pose Landmarker pipeline to extract coordinates of effectors of interest and subsequently analyze kinematic outcomes (related to movements of the hand, shoulder, and trunk). Kinematics of the hand (mean palm speed, palm bivariate variable error; BVE, where a low BVE reflects greater consistency), shoulder (BVE), and trunk (BVE) were extracted for each individual, separately across time points. Exploratory analyses indicate increased mean palm speed and palm BVE across time points. Further, analyses suggest that shoulder and trunk movements may contribute to improvements in hand-related outcomes for some individuals. Overall, our findings provide support for the use of MediaPipe Pose Landmarker in tracking upper limb movements in individuals with motor impairment after stroke.

**Supplementary Information:**

The online version contains supplementary material available at 10.1186/s12984-025-01808-4.

## Introduction

Following stroke, many individuals experience long-term upper limb impairments with less than 20% of individuals demonstrating full recovery by six months after stroke [1]. To develop accessible interventions that promote motor recovery after stroke, there is a need to quantify and track whole arm movement patterns over time. Accurate kinematic data will enable researchers and clinicians alike to discriminate between upper limb functional improvements due to true motor recovery (restitution of patterns seen in age-matched healthy controls) and those stemming from compensatory patterns [2–4]. Thus, development and testing of technologies that permit kinematic movement quantification are needed to complement standardized clinical assessments of sensorimotor recovery post-stroke [3].

While clinical assessments are able to capture changes in post-stroke motor function in terms of task completion, many are not sensitive to *how* the movement is being performed, missing important information detailing movement quality and potentially maladaptive movement patterns that achieve the same functional outcome [3]. Quantifying recovery of movement quality requires high-resolution kinematic data (e.g., the speed and smoothness of movement trajectories and joint angles) to characterize a range of complex movements and changes in movement behaviour. These kinematic variables are traditionally mapped with motion capture systems (e.g., hand position, trunk displacement) (e.g [2, 22]). , . Research-grade motion capture systems can be cumbersome and/or expensive, with some requiring participants to be tethered to the system or use markers that are affixed to the body to track movements using optical, mechanical, magnetic or inertial systems [4]. Advances in computer vision technology have also enabled markerless systems, eliminating the need for physical tracking equipment [5]. Even so, many still rely on system specific equipment, multiple cameras, extensive processing pipelines, and controlled laboratory setups, limiting their practical application in typical clinical environments. Thus, there remains a need for lightweight, high-fidelity, and easily deployable tracking systems suitable for clinical environments.

MediaPipe [6] is an open-source framework for deploying customized machine learning and computer vision solutions. The MediaPipe Pose Landmarker utilizes pre-existing models to map body landmarks on static, recorded or streamed data [7] for human pose estimation [8]. Previous research validated MediaPipe for capturing upper limb movement against inertial-based systems [9], touchscreen computers [10] and standard clinical measurement instruments [11]. MediaPipe has been applied to track gross upper limb movements for joint angle estimation [12], motor skill assessment [13], gait analysis [14, 15], and tremor identification in individuals with Parkinson’s disease [9]. In stroke, MediaPipe has been used for movement quality assessment of the upper limbs compared to Kinect sensors [16]. It has also been applied as a feature extractor for compensation detection using machine learning classifiers [17] and to index proprioception post-stroke [18]. Here, we aim to apply MediaPipe to extract kinematics from single-perspective videos of post-stroke individuals performing a gamified reaching task to identify compensation strategies.

Accordingly, the current proof-of-principle study aimed to determine if MediaPipe Pose Landmarker could track upper limb movements after stroke during a gamified upper limb reaching task using a single camera perspective, and through the extracted kinematics, characterize change in specific kinematic outcomes of interest. To address this objective, individuals with sub-acute and chronic stroke engaged in five sessions of a previously established, semi-immersive, gamified reaching task, involving target-directed movements of the hand and arm [19–21]. Videos that captured hand/arm movements were processed through the MediaPipe Pose Landmarker pipeline to extract coordinates and subsequently analyze key kinematic variables. We expected that coordinates derived from MediaPipe Pose Landmarker data would generate kinematic information that complements clinical outcomes after stroke, including trajectories of the palm, shoulder, and trunk, as well as mean palm speed, allowing both participant and group-level values obtained for each kinematic outcome of interest. As an exploratory aim, we also sought to determine the extent to which shoulder and trunk movements may contribute to hand movements, as past research suggests that individuals with more severe impairment have greater trunk movements, and this may reflect compensatory strategies [22]. Overall, we expected consistency (reflected via a decrease in bivariate variable error; BVE) and mean speed of palm movements to increase across sessions, reflecting increased range of motion when reaching towards a virtual target. We also expected changes in hand-related outcomes (palm BVE and mean speed) to be associated with changes in shoulder and trunk BVE.

## Methods

### Participant data

Seven individuals with sub-acute and chronic stroke (>2 months; [23]) stroke were recruited for the study (aged 75.4 ± 4 years; 4 females). Participants were screened with the Montreal Cognitive Assessment [24] to ensure they could provide consent and follow task instructions and were excluded if they had (1) history of head trauma, seizure, psychiatric diagnosis, neurodegenerative disorder, substance abuse, or other neurological/muscular deficits affecting task performance; or (2) inability to engage in the motor task without arm or shoulder pain. The institutional ethics review board of the University of British Columbia approved the protocol, and all participants gave written, informed consent. Motor impairment was characterized by using the upper extremity portion of the Fugl-Meyer Assessment (FMA-UE, /66; [25]), a stroke-specific, performance-based impairment index, designed to assess motor impairment in individuals with post-stroke hemiplegia, administered and scored by a trained physical therapist. Following assessment, all participants completed five sessions of an unconstrained 3-dimensional motor task [19–21] spaced over a minimum of eight days and a maximum of 14 days. Participant characteristics are included in Table 1.

**Table 1:** Participant characteristics

Participant	Age	Sex	Time since stroke (months)	Hemisphere of stroke	Pre-stroke dominant hand	FMA-UE
1	76	F	57	L	R	60
2	79	F	157	R	R	60
3	73	M	2	L	L(tools) R(writing)	62
4	79	M	128	R	R	60
5	67	F	261	L	R	40
6	77	M	50	R	R	66
7	77	F	157	R	R	63

### Task description

**Fig. 1:**
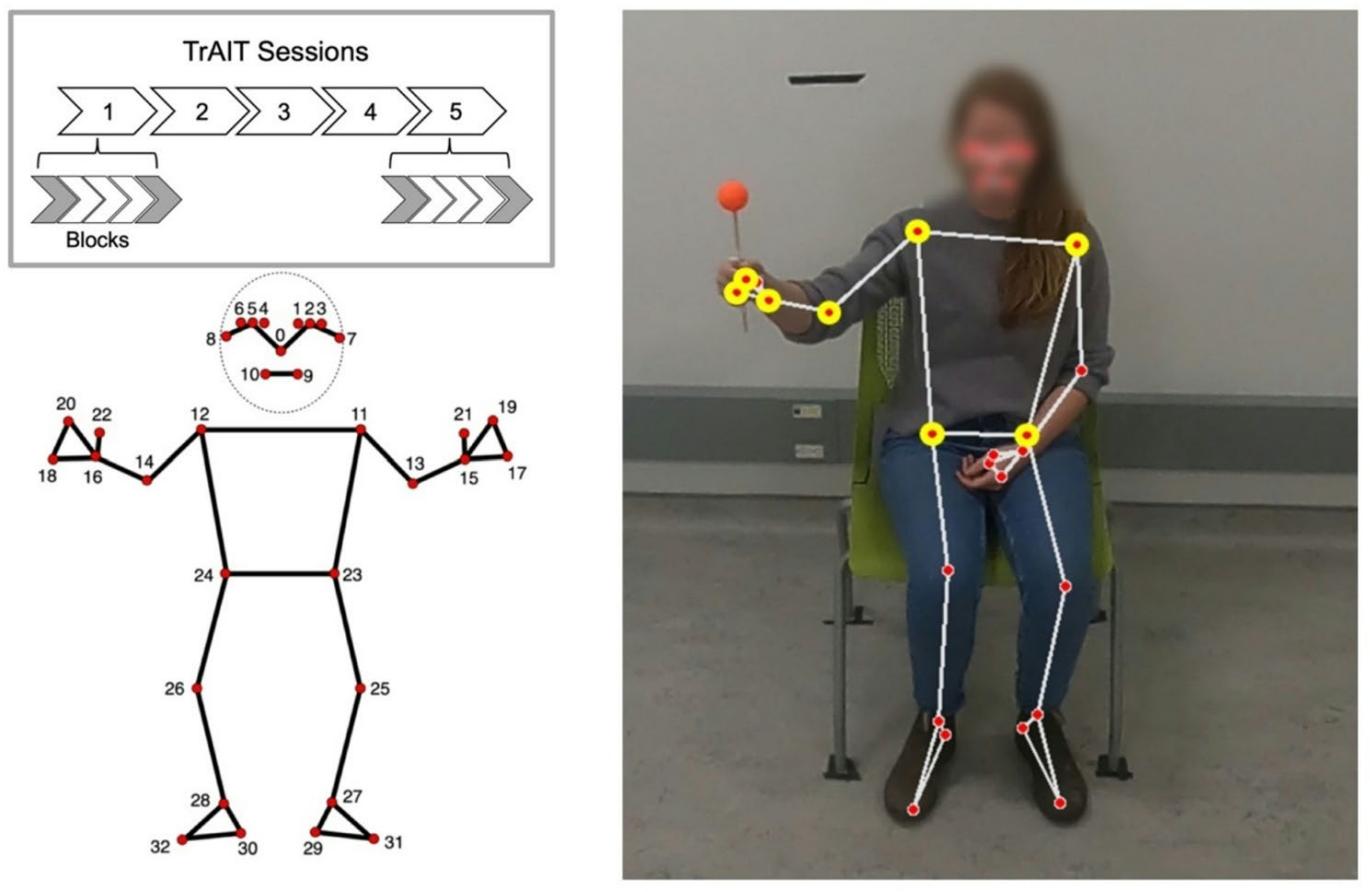
Illustration of the MediaPipe Pose Landmarker predicted skeleton overlaid on a still-image obtained from a video recording of the task with landmarks indicated by red circles with yellow highlights. For illustrative purposes, the individual in this video is holding a spherical object, to contrast with MediaPipe Pose Landmarker predicted landmarks. Markers of interest (8) were located on the pinky finger, index finger, wrist, and elbow of the paretic hand, left and right shoulder, and left and right hip to permit our kinematic analyses. Videos included in analyses from the TrAIT sessions – from the first and last blocks (blocks 1 and 5), for the first and last session (day 1 and 5), shown in grey – are depicted in the grey box

This study used Track and Intercept Task (TrAIT), a semi-immersive virtual reality-based task designed to facilitate motor skill acquisition and improve upper limb motor function after stroke [19–21] (Fig. 1). TrAIT uses a motion-tracking setup that translates participant movements into interactive, real-time gameplay. The task requires participants to move their paretic arm in 3-dimensional space to reach and intercept an asteroid displayed on a screen and throw it toward a target represented by a sun. Missed interceptions fall to the bottom of the screen and ‘explode’. Difficulty level can be customized for the baseline task, and difficulty increases as the participant progresses through levels based on some progression criteria.

### Experimental setup and protocol

A 46-inch monitor was used to display the TrAIT task environment. A Microsoft Kinect camera (model no 1517, Kinect for Windows, 30 FPS; Microsoft, Redmond, WA), affixed on top of the monitor was used exclusively to enable gameplay by tracking patient movements to display elements within the TrAIT environment. Participants were seated 180 cm away from the monitor. The task was calibrated to each participant’s active range of motion in the two-dimensional (2D) plane parallel to the screen (participants were asked to reach as far as they could to all four corners of the screen). The baseline task-level difficulty was tailored to each individual’s capabilities and motor function, by varying (1) interception time allowed per asteroid, (2) asteroid velocity, and (3) asteroid size and target size. To progress to the next level, participants had to achieve a minimum score of 80% (defined as successful interceptions and target hits) on two consecutive blocks [19–21].

All participants engaged in 5 sessions of gameplay each across 5 separate, but not necessarily consecutive days. Each session consisted of five blocks. For each block, participants completed 200 movements with their paretic upper limb (100 interceptions and 100 throws; total of 1000 movements per session). Total session time was approximately 30–40 min. A total of 5000 paretic upper limb movements were performed across the full intervention (2500 interceptions, 2500 throws).

For the purpose of kinematic assessment via MediaPipe Pose Landmarker, we used a GoPro camera (GoPro Hero9; linear field of view 4:3, 1440 resolution, 1920 × 1080, 120fps) mounted on the monitor (i.e., 180 cm horizontal distance from the participant; 170 cm vertical from the floor; angled at approximately 75°) to capture single-perspective, 2D, block-wise videos for each participant). Videos were stored for offline analysis.

### Pose estimation

To address our proof-of-principle objective, we used blockwise videos captured for 7 participants from the first and last blocks (blocks 1 and 5) from the first and last sessions (days 1 and 5) for kinematic assessment. Videos were cropped to exclude any time before the task began and after the task ended, with a mean video duration of 9 min and 2 s. MediaPipe Pose Landmarker, an open-source pose estimation framework from MediaPipe [6], was used for extracting framewise, 2-dimensional position coordinates from participant videos. MediaPipe Pose Landmarker uses BlazePose, a lightweight neural network architecture, for identification of 33 key landmarks on the human body [26]. For our analysis, we extracted 2D coordinates pertaining to eight landmarks - the pinky finger, index finger, wrist, and elbow of the paretic hand, as well as both shoulders and hips of the participant, from each frame in the videos (Fig. 1). We restricted our analysis to 2D space to assess the feasibility of kinematic assessment using single-camera setups. This data was stored for calculating our kinematic outcomes of interest as below.

### Data analyses

#### Primary analysis

To address our main aim, the centre of the palm and centre of the torso (referred to henceforth as ‘palm’ and ‘trunk’) were calculated based on the extracted coordinate data. The palm, defined as the centroid of the pinky finger, index finger, and wrist coordinates, was calculated by averaging the coordinate data for the three known points. Similarly, the trunk, defined as the centroid of the right and left shoulders and hips landmarks were calculated by averaging the coordinate data for the four landmarks. We plotted the movements of the paretic palm, the shoulder, and the trunk for each participant across the four blocks of interest (day 1 - block 1, day 1 - block 5, day 5 - block 1, day 5 - block 5) to visually assess change in performance across sessions.

#### Exploratory analysis

Our exploratory analysis aimed to determine the extent to which shoulder and trunk movements contribute to hand movements post-stroke. Prior work examined movement consistency and mean speeds of the palm, shoulders, and trunk to assess how compensatory movements of the trunk and shoulder relate to palm motion [e.g., 2, 27, 28, 29].

To quantify movement consistency, we calculated **bivariate variable error (BVE)**, a measure commonly used in the motor control literature [e.gs., 30, 31]. BVE captures the summed variation in signed errors across both the x and y directions and was calculated as:

$$\:BVE=\sqrt{\frac{1}{n}\sum\:_{i=1}^{n}{\left(\right({x}_{i}-{x}_{c})}^{2}+{({y}_{i}-{y}_{c})}^{2})\:}$$

To quantify speed, we computed the **framewise speed** of each landmark by dividing the Euclidean distance traveled between consecutive frames by the elapsed time. The **mean landmark speed** was then calculated as the average of all framewise speeds:

For frame f,

$$\:{speed}_{landmark}=\:\frac{\sqrt{{({x}_{f}-{x}_{f-1})}^{2}+{({y}_{f}-{y}_{f-1})}^{2}}}{1/FPS}$$

These measures were calculated for each block and participant. To examine changes across sessions and the potential influence of proximal movements on hand function, we conducted group-level analyses using separate linear mixed-effects (LME) models for palm speed and palm BVE. Fixed effects included Day, Block, trunk BVE, shoulder BVE, and FMA-UE score, with participant modeled as a random intercept.

## Results

### Kinematic outcomes

**Fig. 2:**
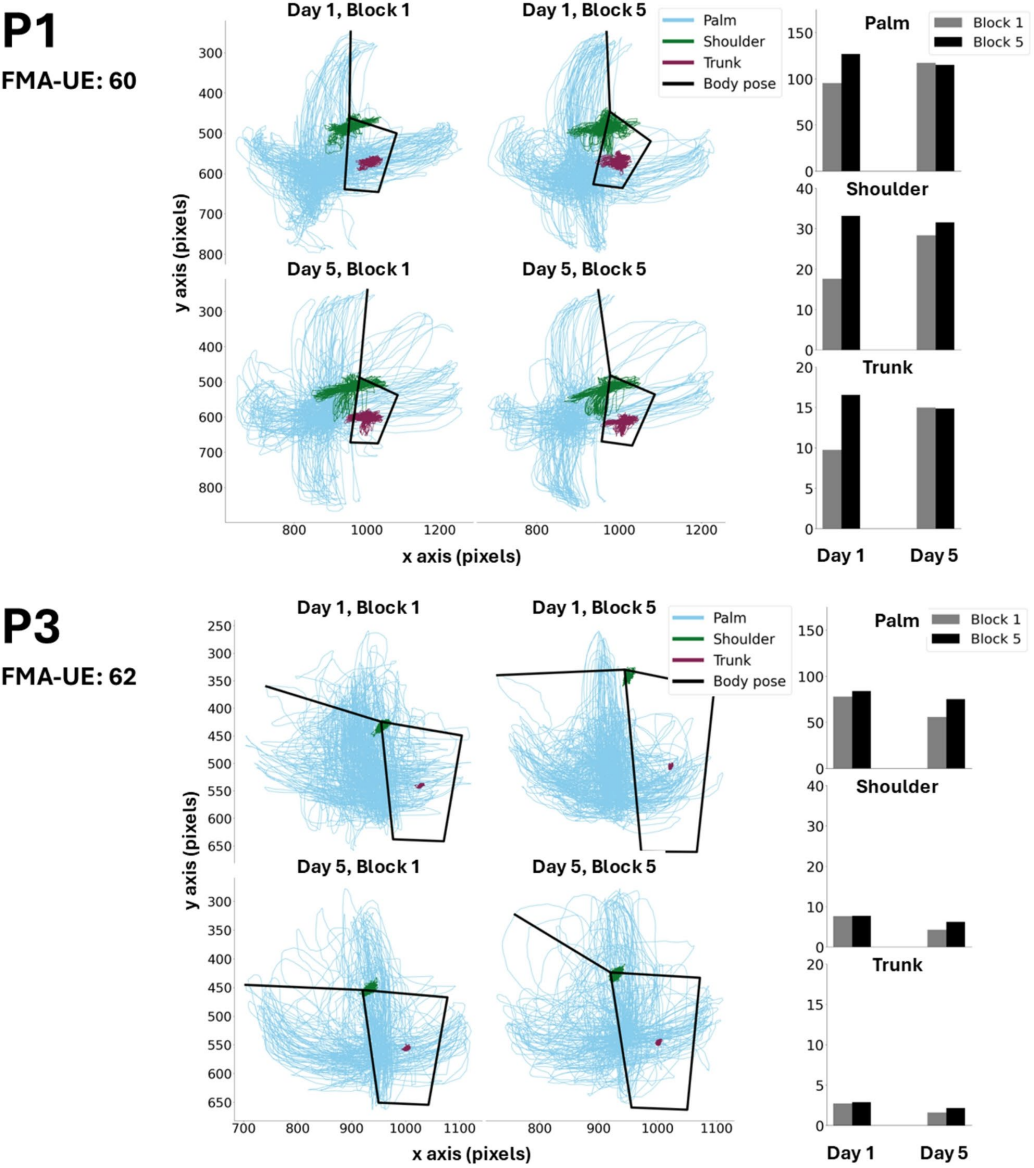
Data from two exemplary participants, including *left*: raw trajectory data, and *right*: bivariate variable error (BVE) calculated from three kinematic outcomes (palm, shoulder, trunk) obtained from the paretic limb for the first and last block and day of practice. The black shape represents each participant’s initial trunk position, outlined by the left and right shoulder, and the left and right hip. BVE was calculated using continuous position data across each block. (**P1**): from visual inspection of the raw trajectory data and bar graphs, P1 appears to display substantial movements of the trunk and shoulder during the task across blocks/days. BVE of the shoulder and trunk also appeared to increase across blocks/days. **(P3)**: from visual inspection of the raw trajectory data and bar graphs, P3 appears to display minimal movement of the trunk and shoulder, relative to palm movements during the task. BVE of the shoulder and trunk also appeared consistent across practice

Mean BVE, calculated across palm and shoulder (obtained from the paretic limb) and trunk, is reported in Table 2. Raw data of predicted marker positions for the palm, shoulder, and trunk for two exemplar participants are shown in Fig. 2. BVE of the palm and shoulder calculated across position data separately for each block are also shown for two exemplar participants in Fig. 2. BVE of the paretic hand increased across practice (Day 1, Block 1 vs. Day 5, Block 5) for both participants (i.e. consistency decreased). However, two different patterns of BVE in shoulder and trunk movements accompanied changes related to the hand. In the second participant (Fig. 2; P3, BVE of the shoulder and trunk appeared to remain consistent with practice across sessions. In contrast, based on visual trends observed in the bar graphs in Fig. 2, BVE for the shoulder and trunk of the first participant (Fig. 2; P1) appeared to increase with practice on Day 1 and was maintained at Day 5.

**Fig. 3:**
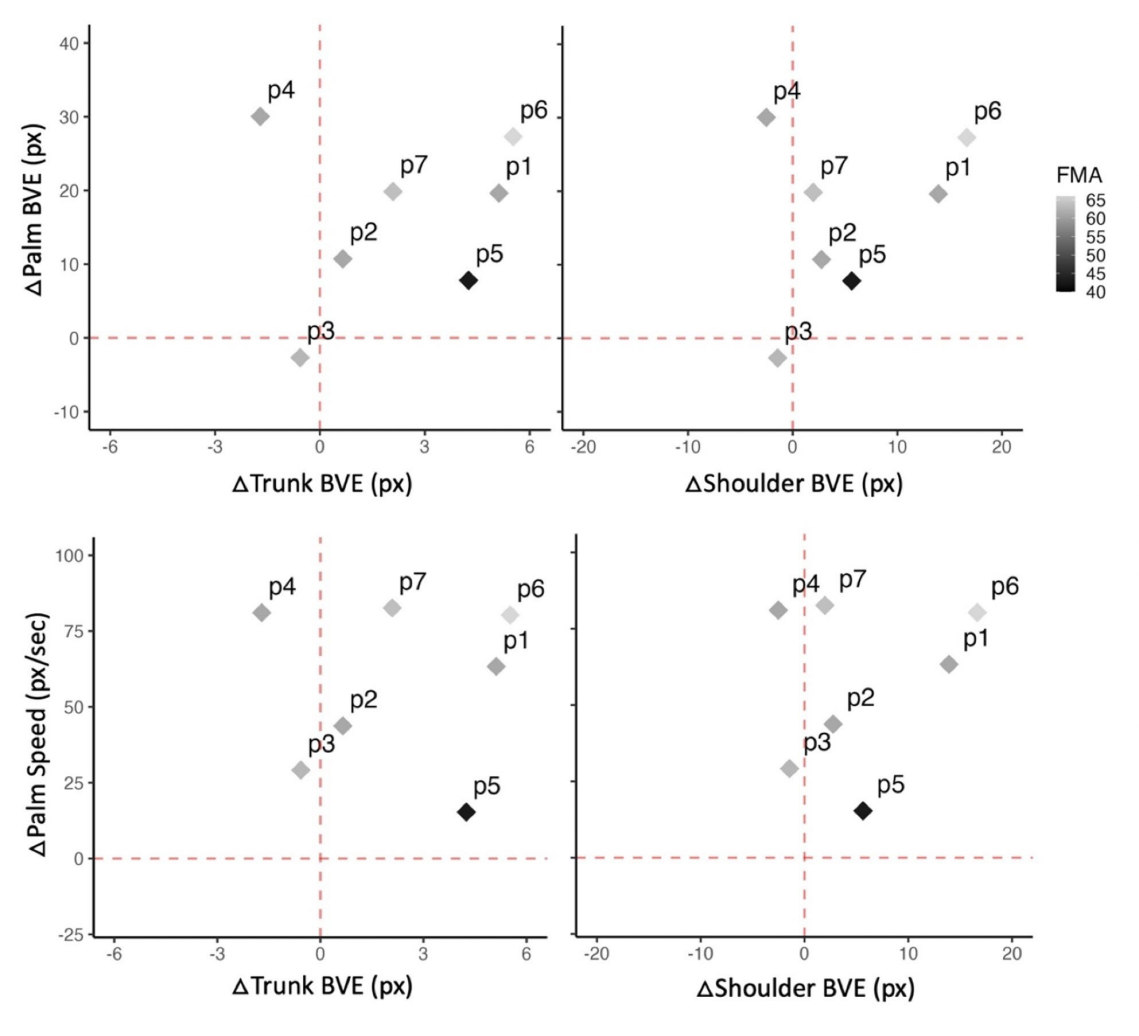
Changes in hand outcomes (palm speed, palm BVE) is shown against change in trunk (top) and shoulder (bottom) BVE for each participant. Fugl-Meyer score (FMA-UE; /66) represented by colour shading of each participants data point, with darker points representing lower FMA-UE scores (i.e., greater motor impairment). Change of each outcome measure was calculated for each participant as Day 5 Block 5 minus Day 1 Block 1 (i.e., last block minus first block of practice). The dashed lines represent positive and negative change for each outcome measure. For example, the top right quadrant indicates increases for all outcome measures (indicating greater variability of movements), whereas the top left quadrant indicates increased hand outcomes and decreased trunk/shoulder BVE (indicating greater consistency in trunk and shoulder movements across practice)

Figure 3 shows potential associations across all participants between change in palm kinematics (change in palm BVE and mean speed) and both change in trunk BVE (Fig. 3; top), and change in shoulder BVE (Fig. 3; bottom). Change was calculated as the last minus first block of practice (i.e., Day 5 Block 5, minus Day 1 Block 1). Overall, hand outcomes were generally positive: palm speed appeared to increase across practice. This increase was associated with decreases in shoulder and trunk BVE for two participants and associated with increases in shoulder and trunk BVE for five participants. Palm BVE appeared to increase for six participants, yet this increase was only accompanied by a decrease in shoulder and trunk BVE for one participant (Fig. 3).

### Exploratory analyses

Table 3 reports our two exploratory analyses conducted to consider interactions between hand outcomes and trunk and shoulder movements, as well as motor impairment (measured by the FMA-UE). The LME model conducted on mean palm speed revealed that at a group level there was a significant effect of Shoulder BVE (*β* = 7.78, *p* < .001), but not Trunk BVE, and no interaction between these factors (all *p-values* > 0.05). As shoulder mobility increased, palm speed increased. In addition, there was also a significant effect of FMA-UE score (*β* = 5.03, *p* < .001), whereby individuals with higher FMA-UE scores (less motor impairment), had faster palm speeds. Accounting for these factors revealed an effect of Day for mean palm speed (*β* = -17.21, *p* = .008). Mean palm speed was significantly faster on Day 5 than on Day 1 when performing TrAIT. However, there was no effect of Block, nor an interaction between Day and Block (*ps* > 0.05; Table 3).

For the LME conducted on Palm BVE, there was a significant interaction between Trunk and Shoulder BVE (*β* = − 0.13, *p* = .005). Here the effect of trunk movements on Palm BVE was reduced with increased shoulder movement. Furthermore, inclusion of FMA-UE scores revealed that participants with less motor impairments had greater palm BVE (*β* = 2.32, *p* = .014). Accounting for these variables revealed a significant main effect of Day (*β* = -6.52, *p* = .014). There was greater Palm BVE on Day 5 compared to Day 1. There was no main effect of block, or an interaction between Day and Block (*ps* > 0.05; Table 3).

**Table 2:** *Mean (and SD) values for each kinematic outcome across blocks and sessions*

	Day 1		Day 5	
	Block 1	Block 5	Block 1	Block 5
BVE palm	97.1(25.5)	97.6(32.1)	118.6(38.1)	113.2(35.4)
BVE shoulder	20.4(11.2)	19.9(11.1)	27.0(13.4)	25.7(12.7)
BVE trunk	10.2(5.2)	9.2(5.5)	13.4(7.4)	12.5(6.9)
Mean palm speed	123.3(31.1)	140.2(52.5)	179.9(58.5)	179.9(54.6)

**Table 3:** Linear mixed effects models conducted to assess changes in palm BVE and mean palm speed

	Palm BVE			Palm speed (mean)			
*Predictors*	*Estimates*	*CI*	*p*	*Estimates*		*CI*	*p*
(Intercept)	-91.18	-194.85–12.50	0.077	-243.05		-378.66 – -107.45	**0.002**
Day [Day 1 vs. Day 5]	-6.52	-11.35 – -1.69	**0.014**	-17.21		-28.58 – -5.83	**0.008**
Block [Block 1 vs. Block 5]	1.04	-1.07–3.15	0.308	-3.85		-9.24–1.54	0.148
Trunk BVE	1.53	-1.39–4.44	0.285	-2.26		-9.09–4.58	0.502
Shoulder BVE	3.70	1.84–5.57	**< 0.001**	7.78		4.16–11.39	**< 0.001**
FMA-UE	2.33	0.61–4.04	**0.014**	5.03		2.85–7.21	**< 0.001**
Day [Day 1 vs. Day 5]*Block [Block 1 vs. Block 5]	-1.79	-3.86–0.28	0.085	-3.77		-9.16–1.63	0.156
Trunk BVE*Shoulder BVE	-0.13	-0.21 – -0.04	**0.005**	-0.16		-0.33–0.02	0.080
**Random effects**							
σ^2^	23.59				162.36		
τ_00 Participant: Day_	31.37				158.83		
τ_00 Participant_	167.81				93.05		
ICC	0.89				0.61		
N _Participant_	7				7		
N _Day_	2				2		
Observations	28				28		
Marginal R^2^ / Conditional R^2^	0.723 / 0.971				0.844 / 0.939		

## Discussion

As a proof of concept, we evaluated whether MediaPipe Pose Landmarker could successfully track upper limb reaching movements after stroke during a gamified task and characterize change of specific kinematic outcomes of interest. Participants with upper limb motor impairment after stroke completed five sessions of gamified reaching task that was video recorded using a single camera perspective. Position data of the shoulder, hand, and trunk were estimated from the video recordings using an open-source, full-body pose estimation solution (MediaPipe; [6]). Subsequently, we calculated consistency (BVE) of the shoulder, trunk, and hand, as well as mean palm speed. Here, we provide evidence in support of this approach; kinematic data was successfully extracted for all participants; some individuals showed an increase in shoulder and trunk BVE across sessions, and shoulder movements were shown to increase with improved hand-related outcomes (shown as shoulder BVE increased with palm BVE). Below we discuss these findings in the context of past work testing markerless approaches and movement patterns after stroke.

Past work has evaluated a variety of markerless approaches via Kinect and RGB cameras for identifying compensatory strategies through video-based pose estimation [32, 33]. Foreman and Engsberg [33] tested Kinect-derived kinematic measures of trunk compensation against an eight-camera video motion capture system, reporting high correlations between assessment tools for measures of both trunk and lateral flexion. While we did not compare our approach to a known standard kinematic assessment tool, past work evaluating the use of MediaPipe against other tools for assessing upper limb movement kinematics has shown good alignment with trajectories predicted by MediaPipe with those captured by other motion capture systems (e.g. Kinect, touchscreen computer; [10, 16]). This reinforces its use as a cost-effective, single camera approach as compared to other resource intensive systems to track and inform motor recovery after stroke. As this approach provides kinematic assessment, other open-source software like OpenCap [34], which require two or more camera views, can be leveraged to provide additional information regarding the dynamics of upper limb movement in future work, and may serve as alternative approaches depending on the use case.

### Clinical relevance

Murphy and Hager [35] demonstrated that individuals with upper limb hemiparesis exhibit slower and less accurate movements when compensatory strategies are employed, in-turn recommending both the use of task-related (i.e., accuracy, movement time) and effector-based (i.e., kinematic) measures for evaluating upper extremities after stroke [3]. Previous work employing the TrAIT has demonstrated improvements in performance marked by decreased movement time and faster rate of skill acquisition across practice [19–21]. However, it is not clear what kinematic patterns individuals used to achieve these performance gains. Others have suggested that key metrics could be extracted to delineate strategies being employed during skilled motor practice after stroke [36], including trunk and shoulder movements, which have been shown to be an important marker of compensatory strategies [e.g., 29, 36]. For instance, increased trunk displacement and decreased peak velocity were also noted in individuals after stroke with motor impairment during reach-and-grasp tasks [36]. Other work has attributed performance improvements on a reaching task to an increased activation of the trunk muscles (i.e., rather than to skill acquisition *per se*; [37]). In line with this work, our exploratory analyses suggest that increasing shoulder movements contributed, in part, to observed increases in both palm-related outcomes. Further, consistency of trunk movements increased (i.e., decreased trunk BVE) for some individuals, and contributed to palm-related outcomes. However, the effect of trunk movements was reduced as shoulder movements increased. While we can only speculate, some participants may have instead been able to improve function with increased degrees of freedom of the shoulder rather than rely on compensatory movements of the trunk to transport their hand to intercept the target. Thus, our work supports the addition of effector-based kinematics (i.e., in addition to endpoint related outcomes as reported in past work [19–21]) towards providing greater resolution about movement strategies or the quality of movement that is achieving the task outcome (e.g.s [2, 22, 37, 38]), particularly when engaging in skilled motor practice of a reaching task after stroke.

### Limitations and future directions

It is important to consider that our investigation examined the use of MediaPipe Pose Landmarker to track 2D trajectories of a 3D upper-limb reaching task after stroke. Testing this approach represents a necessary step towards establishing a cost-effective approach to quantifying movement in clinical or community settings (e.g., home-based). For instance, past work suggests the possibility of using 2D MediaPipe to automate FMA-UE for telerehabilitation [39]). Yet, assessing movements in three dimensions is integral to quantifying motor function and/or level of motor impairment. Future work testing the use of MediaPipe and other artificial intelligence-based pose estimation systems for 3D tracking that include depth information will be critical to the automation of assessments of upper limb motor function.

Given our main aim, we did not include a control group. The sample in this proof-of-concept study was designed to characterize within-participant change and was limited to individuals with mild motor impairment. While our ability to attribute observed changes specifically to the skilled motor practice is thus limited, it is unlikely that changes observed here were due to familiarization as TrAIT is designed to adapt to an individual’s capabilities (i.e., decreasing target size and increasing target speeds [19–21]), and neural, behavioural, and functional outcomes driven by TrAIT have been demonstrated in past work [19–21]. However, future work testing movement patterns across a larger sample of individuals with severe motor impairment is needed to strengthen the applicability of this tool in a clinical setting.

In conclusion, we tested the use of an artificial intelligence-based markerless pose estimation system (MediaPipe Pose Landmarker) in tracking upper limb reaching movements in individuals with motor impairment after stroke. Our findings provide support for this approach, complementing standardized clinical measures to characterize change in movement quality after repetitive arm movement training; we captured relative position data of the palm, shoulder, and trunk across five sessions of skilled motor practice in individuals after stroke. Exploratory analyses revealed improvements in hand-related outcomes across practice were associated with increased shoulder and trunk movements, demonstrating how kinematic outcomes can contextualize movement strategies as reported in past work [2, 22, 37, 38]. Overall, this work represents an important step towards implementing open-source and artificial intelligence-based software in both research and clinical settings to evaluate improvements in performance and/or function driven by skilled motor practice after stroke.

## Supplementary Information

Below is the link to the electronic supplementary material.

Supplementary Material 1.

## Data Availability

Study data are not openly available due to reasons of sensitivity and are available from the corresponding author upon reasonable request.
