# Supplementary material for: Using MediaPipe to track upper-limb reaching movements after stroke: a proof-of-principle study
Source: J Neuroeng Rehabil. 2025 Nov 25;22:268. doi: 10.1186/s12984-025-01808-4 (PMC12752325; doi:10.1186/s12984-025-01808-4)
Supplement: Supplementary file 1 — Supplementary Material 1. [file 12984_2025_1808_MOESM1_ESM.docx]

Supplementary Materials


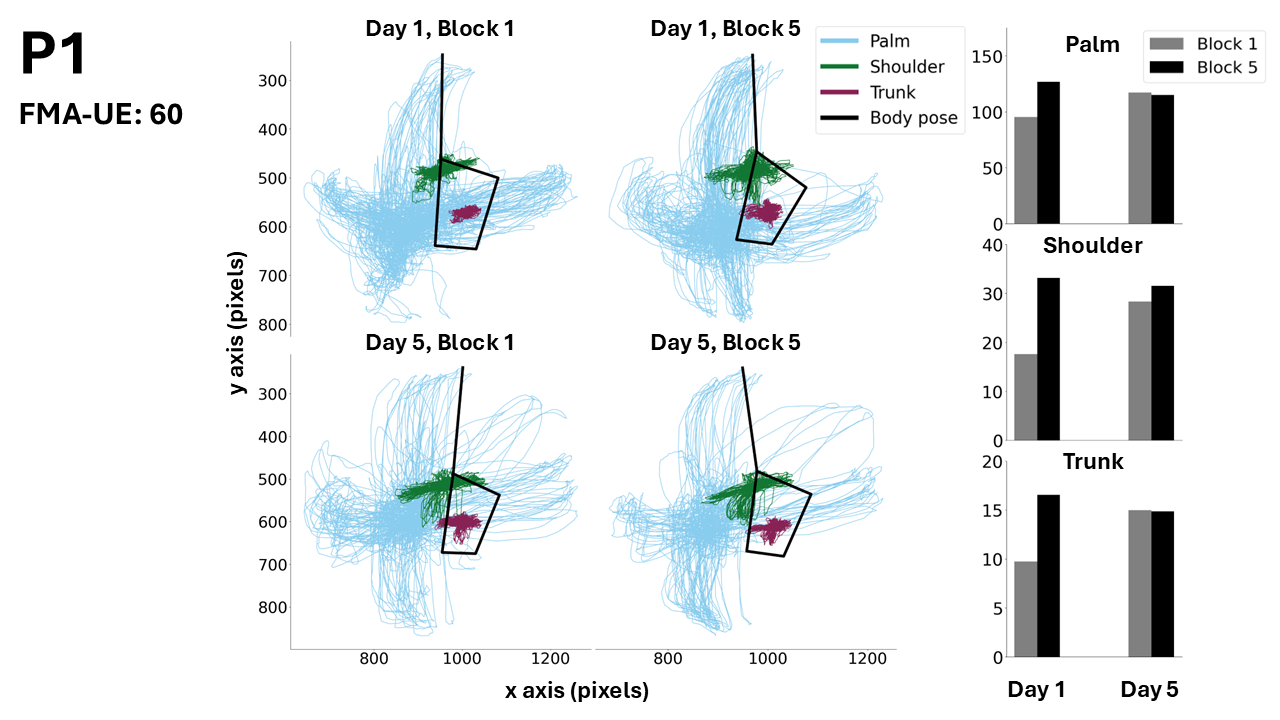

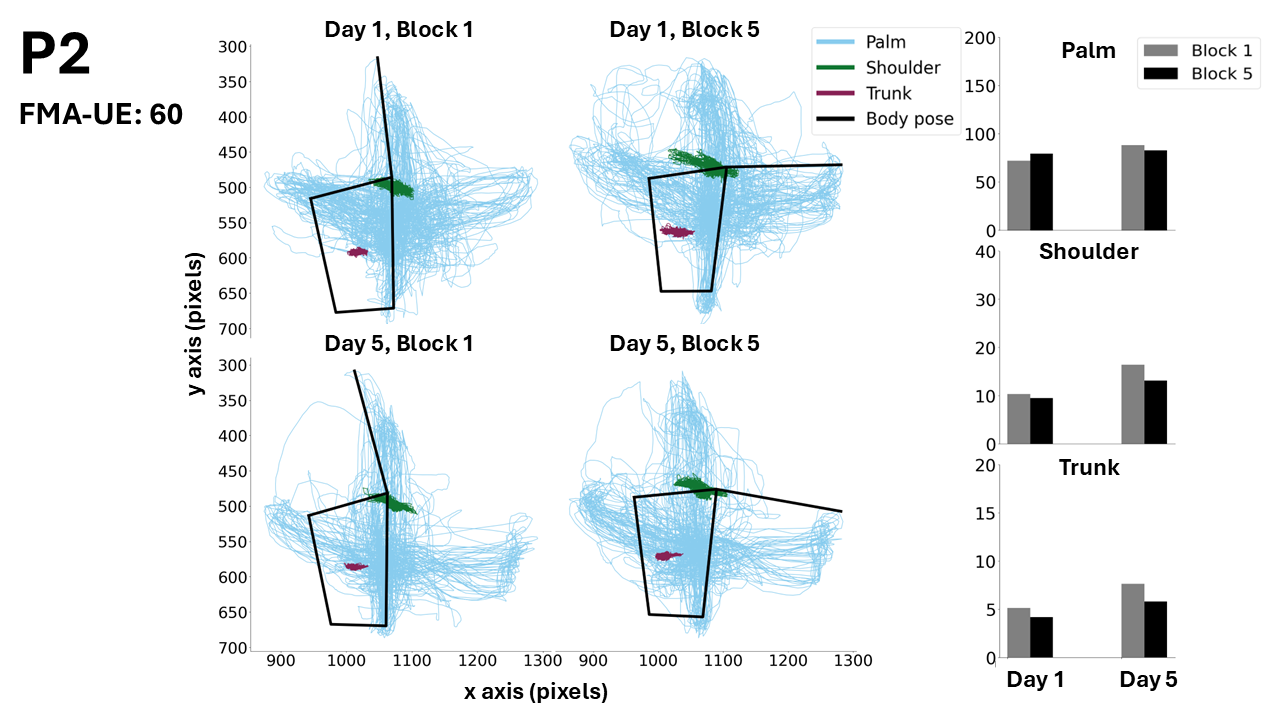

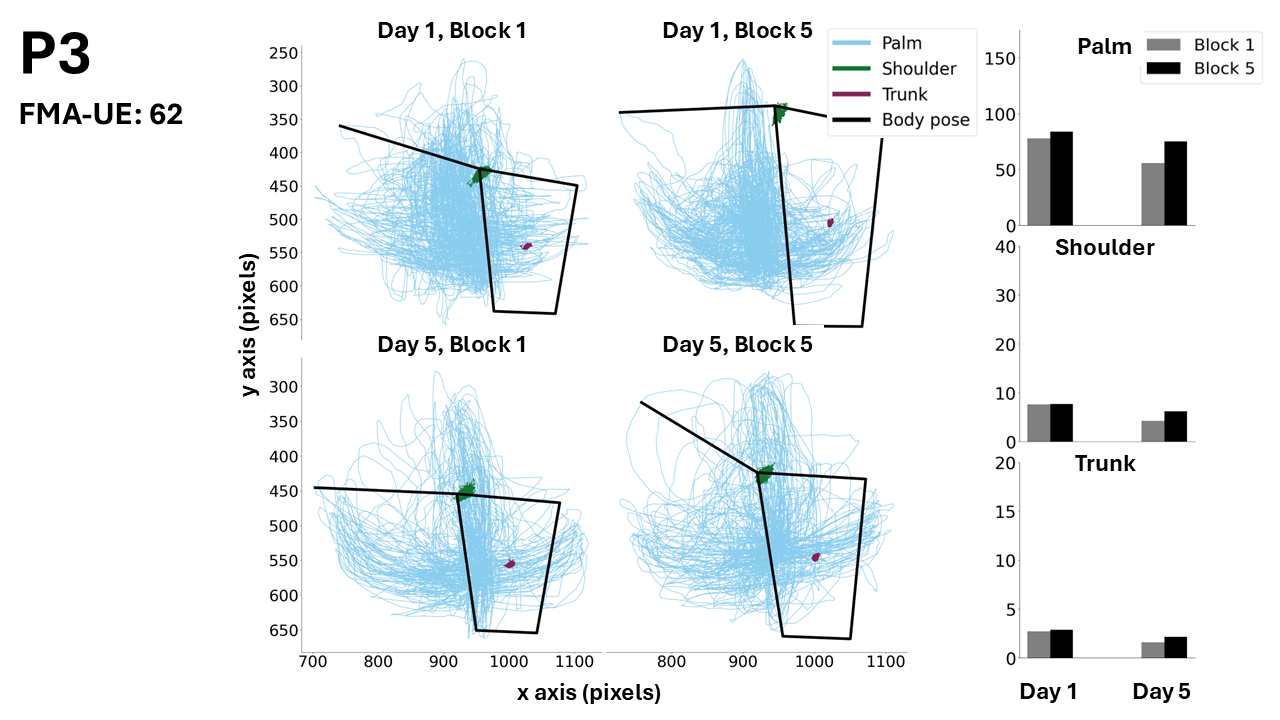

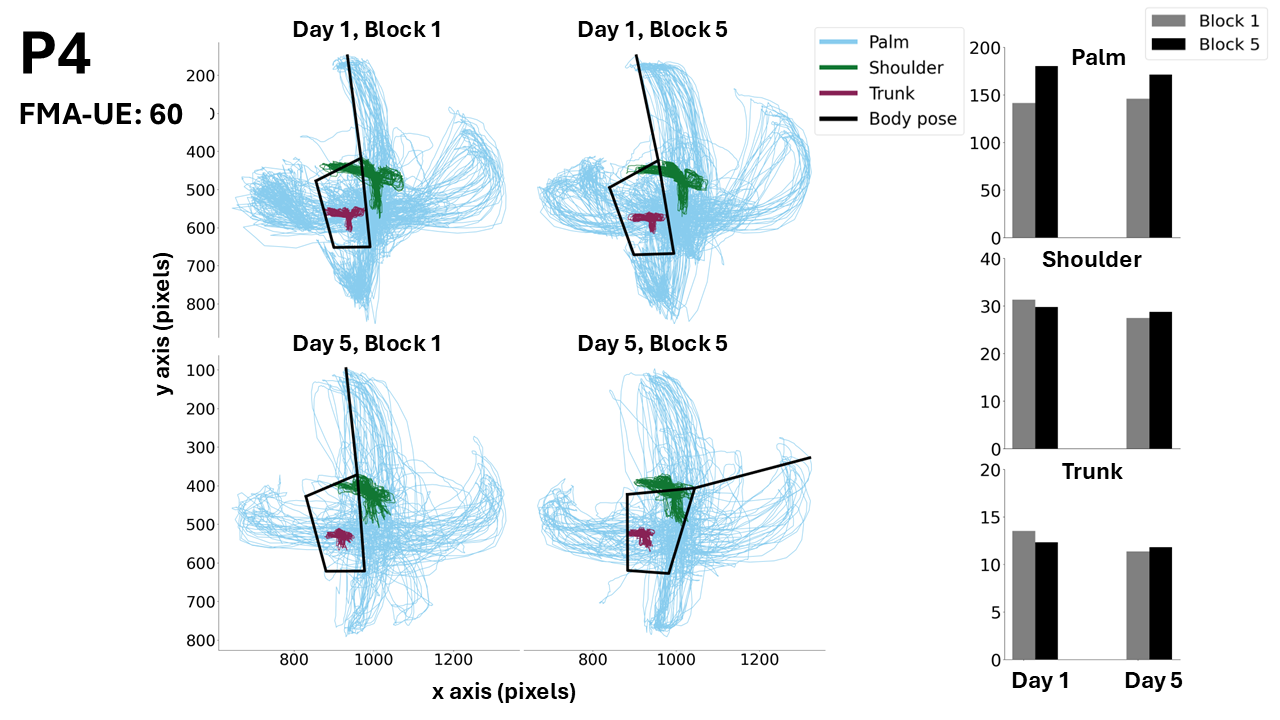

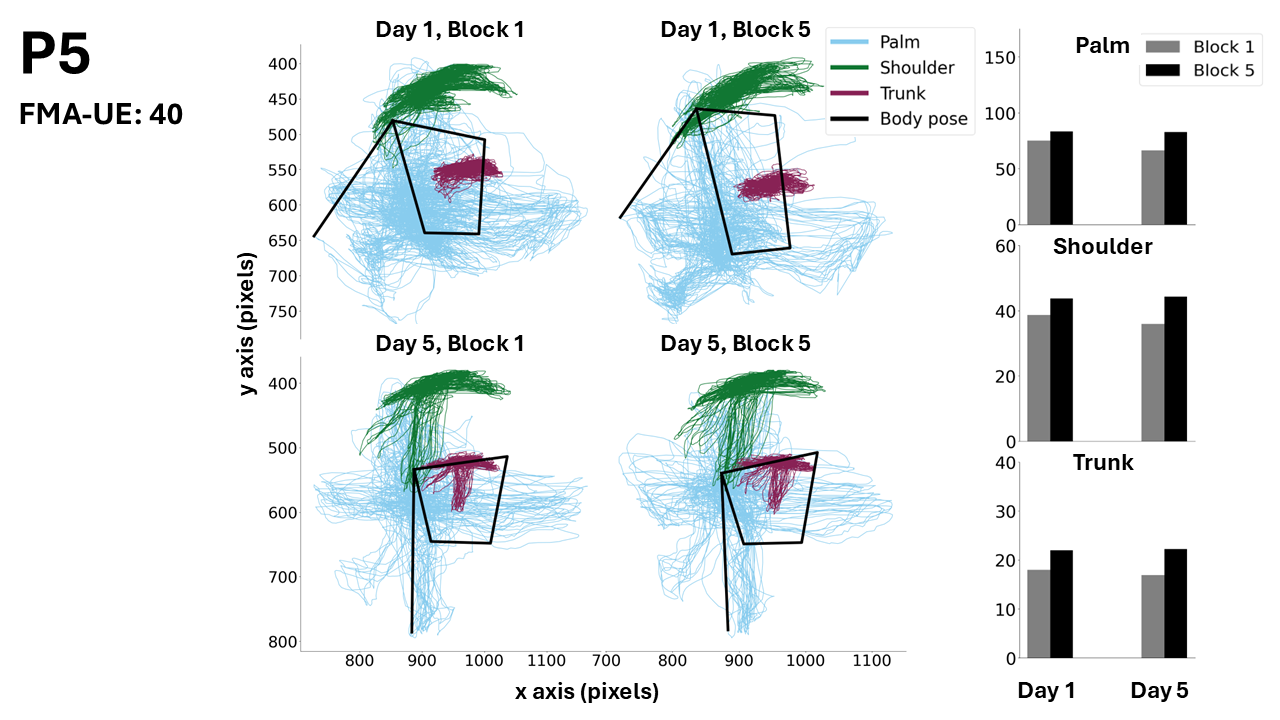

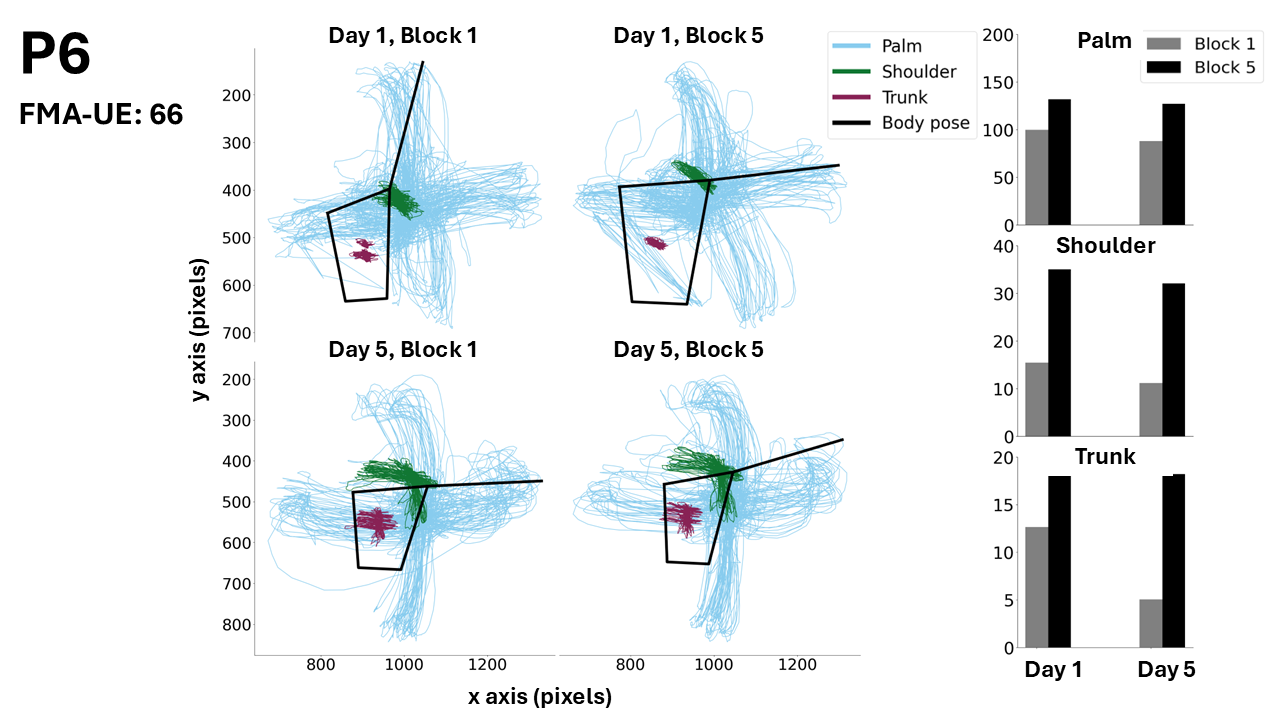

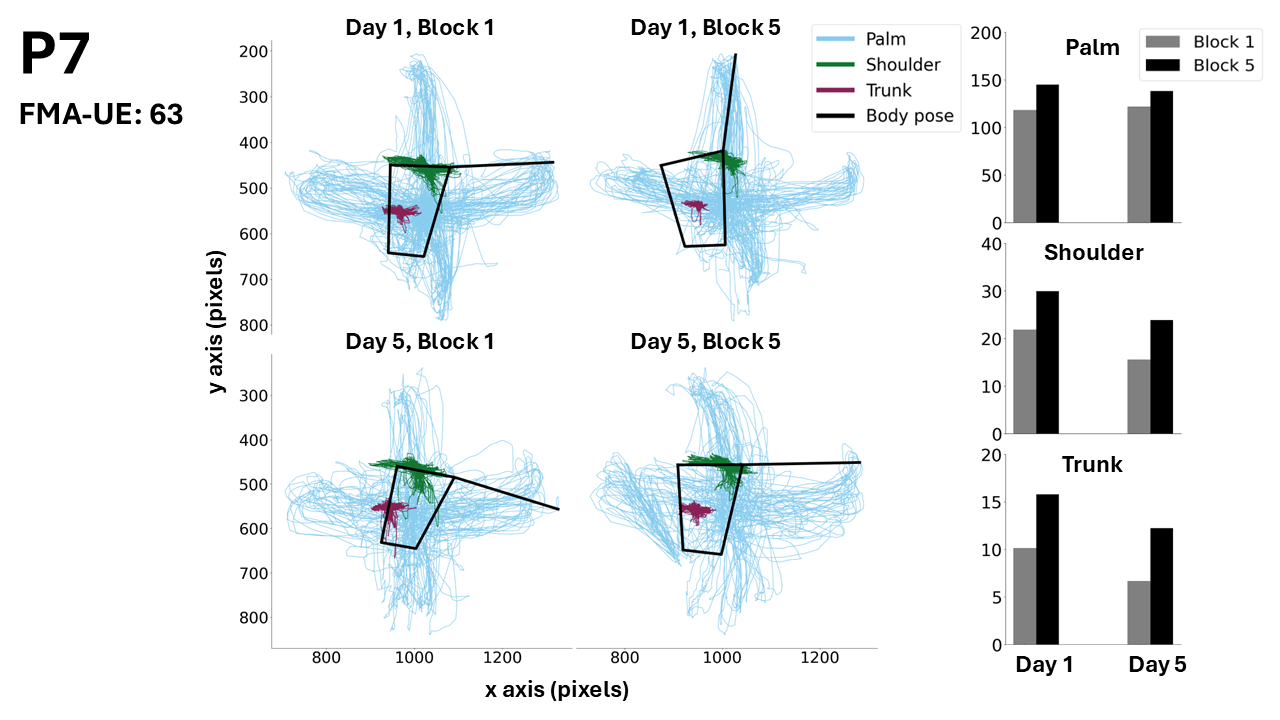


*Supplementary Figure 1.* Data from all participants, including left: raw trajectory data, and right: bivariate variable error (BVE) calculated from three kinematic outcomes (palm, shoulder, trunk) obtained from the paretic limb for the first and last block and day of practice. The black shape represents each participant’s initial trunk position, outlined by the left and right shoulder, and the left and right hip. BVE was calculated using continuous position data across each block.
